# A Clinically Integrated Post-Graduate Training Programme in Evidence-Based Medicine versus ‘No Intervention’ for Improving Disability Evaluations: A Cluster Randomised Clinical Trial

**DOI:** 10.1371/journal.pone.0057256

**Published:** 2013-03-01

**Authors:** Rob Kok, Jan L. Hoving, Paul B. A. Smits, Sarah M. Ketelaar, Frank J. H. van Dijk, Jos H. Verbeek

**Affiliations:** 1 Coronel Institute of Occupational Health, Academic Medical Center, Amsterdam, The Netherlands; 2 Research Center for Insurance Medicine, Academic Medical Center, Amsterdam, The Netherlands; 3 Cochrane Occupational Safety and Health Review Group, Finnish Institute of Occupational Health, Kuopio, Finland; Penang Medical College, Malaysia

## Abstract

**Background:**

Although several studies have shown that teaching EBM is effective in improving knowledge, at present, there is no convincing evidence that teaching EBM also changes professional behaviour in practice. Therefore, the primary aim of this study was to evaluate the effectiveness of a clinically integrated post-graduate training programme in EBM on evidence-based disability evaluation.

**Methods and Findings:**

In a cluster randomised controlled trial, fifty-four case-based learning groups consisting of 132 physicians and 1680 patients were randomly assigned to the intervention or control groups. A clinically integrated, post-graduate, 5-day training programme in evidence-based medicine, consisting of (home) assignments, peer teaching, interactive training in searching databases, lectures and brainstorming sessions was provided to the intervention group. The control group received no training. The primary outcome was evidence-based disability evaluation, as indicated by the frequency in use of evidence of sufficient quality in disability evaluation reports. There are no general EBM behaviour outcome measures available. Therefore, we followed general guidelines for constructing performance indicators and defined an a priori cut-off for determination of sufficient quality as recommended for evaluating EB training. Physicians trained in EBM performed more evidence-based disability evaluations compared to physicians in the control group (difference in absolute proportion 9.7%, 95% CI 3.5 to 15.9). The primary outcome differences between groups remained significant after both cluster-adjusted analysis and additional sensitivity analyses accounting for subjects lost to follow-up.

**Conclusions:**

A EBM programme successfully improved the use of evidence in a non-hospital based medical specialty. Our findings support the general recommendations to use multiple educational methods to change physician behaviour. In addition, it appeared important that the professional context of the intervention was very supportive in the sense that searches in databases, using and applying guidelines and other forms of evidence are considered standard practice and are encouraged by colleagues and management.

## Introduction

Evidence-based medicine (EBM) is widely recognised as a useful tool for improving the quality of health care by supporting clinical decision-making [1], [2]. Moreover, the use of evidence is considered good clinical practice [3]. The EBM approach has also become increasingly acknowledged in non-hospital settings, such as rehabilitation and public health [4], [5]. For insurance medicine, we showed in a previous study how the quality of decision-making in disability evaluation can be improved using evidence [6].

Worldwide, disability evaluations are performed by physicians, either in addition to other clinical work or as one of their main tasks. Although the settings, insurance and legislative systems, and clinical backgrounds are different in each nation, all disability evaluations have *in common* the use of medical and non-medical information, as described in the WHO-ICF classification, and include a judgment of an individual's functioning or capacity to perform work [7]. In the Netherlands, social-insurance physicians employed by the Dutch National Institute for Employee Benefit Schemes perform disability evaluations. These physicians systematically evaluate whether workers who apply for a disability benefit are impaired in one or more mental or body functions due to health problems. The evaluation also includes an assessment of the prognosis and of the therapeutic and return to work options. To date, most of these evaluations are based on expert judgment. However, because expert judgment is known to be prone to biases [8], these evaluations ought to be underpinned with up-to-date information from studies on work disability, diagnosis, treatment effectiveness, and prognosis [6].

Although several studies have shown that teaching EBM is effective in improving knowledge, at present, there is no convincing evidence that teaching EBM also changes professional behaviour in practice or improves health care outcomes. The evidence is especially lacking in the field of disability evaluation. In a systematic review by Coomarasamy and Khan in 2004, two Randomised Controlled Trials (RCTs) of post-graduate teaching in EBM integrated with clinical practice revealed improvements in physicians' behaviour, whereas standalone teaching was not effective [9]. Since then, three other RCTs [5], [10], [11] of post-graduate EBM training have been performed, of which one showed an enhanced use of evidence by physicians [11] Two other trials among public health and primary care physicians did not identify an effect on professional behaviour [5], [10]. These results show that there is still a lack of evidence of the effect of post-graduate teaching of EBM on physician behaviour.

Recently, we developed an EBM course that included training material to improve evidence-based disability evaluation. In a pilot study, we showed that a short training session improved physicians' knowledge and skills in using medical evidence [12], but we did not measure physician behaviour. We then developed a comprehensive, multifaceted and clinically integrated post-graduate training programme in EBM. We paid special attention to educational (not specifically teaching the EBM method) intervention components known to be successful in influencing physician behaviour, such as high attendance, mixed interactive and didactic sessions, use of multi-media, multiple exposures, needs assessment and small-group learning [13]–[15]. By modelling these components, we aimed to motivate the physicians to incorporate evidence from scientific research in their decision-making and to use this evidence in their disability evaluation reports, which we termed evidence-based disability evaluation.

We evaluated this intervention in a cluster-randomised controlled trial of physicians working for the Dutch National Institute of Benefit Schemes. These physicians were members of a network of case-based learning groups. Teaching members of these groups has the potential advantage that available organisational structures are utilised and that knowledge and skills can be disseminated easily by physicians amongst colleagues in their groups. For the above reason but especially because we used multiple reports from each physician which leads to probable clustering at the level of physicians, we chose a cluster-randomised controlled study design to control for this The primary aim of this trial was to evaluate if a clinically integrated post-graduate training programme in EBM compared with no training leads to more evidence-based disability evaluation. The secondary aim was to evaluate if this training programme improves several intermediate factors, such as knowledge, skills and self-efficacy, in the EBM method.

## Participants, Intervention and Methods

### Design

We used an assessor-blinded, cluster-randomised controlled design with two arms: a group of physicians from case-based learning groups who received our clinically integrated post-graduate training in EBM and a waiting list control group from case-based learning groups who practiced as usual. Equal allocation ratio was applied. Ethics approval for this study was sought from the research ethics committee of the Academic Medical Center. However, as this constituted the evaluation of an educational intervention with physicians, the committee secretary deemed it could be suitably exempt from the need for ethics approval. This exemption was confirmed in writing.

### Sample size calculation

For the power calculation, made in advance, we made the following assumptions for the effect size: we assumed that we could increase our primary outcome, the use of evidence in disability evaluation, by 80%. We based this on Kok et al. [12] who showed an increase of 80% of the baseline rate in the use of PubMed after an introductory EBM course in disability evaluation. The control rate of the use of evidence was estimated, based on the same study, at 6.7% of the disability evaluations. This leads to an assumed intervention group rate of slightly above 12%. To have 80% power to detect an increase in the use of evidence of 80% with alpha  = 0.05, we needed 465 disability evaluations in each group. We assumed the cluster effect would be strongest for the disability evaluation reports at the physician level and that the effect of being in the same case-based learning group would be negligible. We assumed that each physician would contribute 10 disability evaluations to the study on average and that there would be an intra-cluster correlation coefficient of 0.1, as determined in implementation research [16]. Based on these data, we multiplied the sample size with an inflation factor of 1.9 to adjust for the cluster effect [17]. This led to a required sample size of 884 disability evaluation reports in each group or 88 physicians. With three physicians per cluster, we needed 30 case-based learning groups in each arm of the trial.

### Participants

Participants were selected from approximately 700 physicians working for the Dutch National Institute of Benefit Schemes. The task of these physicians is to evaluate the disability of workers who apply for a disability benefit. Their evaluation is written down in a disability evaluation report. Besides this report, where the substantiating of their evaluation takes place, and which is considered the main output for these physicians, they also use a checklist with the most important mental and body functions and rate the impairments due to the health problem presented by the patient (see Appendix S1). This checklist is comparable with the Personal Capacity Assessment in the United Kingdom [18]. The impairment rating is subsequently used by labour experts who then assess if and to what extent these impairments lead to a theoretical loss of earnings. Based on this assessment, the patient is then granted or denied a disability benefit.

All of these physicians have to take part in so-called case-based learning groups, located in all regions in the Netherlands, with an average of eight to ten colleagues, with the aim to increase the quality of professional performance. Leaders from these groups invited two to three physicians per group as volunteers to participate in the study. The physicians were informed about the goal of the study and the random assignment of the case-based learning groups to either the intervention or the control group. Except for belonging to a case-based learning group, no further eligibility criteria were used. All participating physicians signed an informed consent form. All groups had agreed to participate before randomisation took place.

### Randomisation and blinding

The case-based learning groups were the unit of randomisation. Randomisation was performed by an independent researcher who was not involved in the study and was provided with a sequentially numbered list of 54 case-based learning groups, to ensure blinding for participants of each group. With the computer program nQuery Advisor (nQuery Advisor® 6.0), he produced a random list of intervention or control assignments based on a mixed-block (size 4) sequence and pre-stratification in three strata based on group size. Except for group size, no further pre-stratification variable was used. The independent researcher applied these assignments to the list of groups and provided the result to a research assistant not involved in scoring. Subsequently, the research assistant invited participants to the training or control condition groups, and changes to the participants list were not allowed.

Two independent assessors blind to treatment allocation scored the outcome measures that were not based on self-assessment, including the primary outcome (evidence-based disability evaluation) and the Fresno Test of Evidence-Based Medicine. Obviously, no physicians in either group were blinded.

### Intervention

On the basis of earlier experience and needs assessment [12], we developed a comprehensive, multifaceted and clinically integrated EBM educational programme to teach the use of all EBM steps in the context of disability evaluation. The course included 5 contact days over a six-month period, with multiple exposures to the EBM method. During the programme, feedback was provided in between course days, a component considered important in changing the behaviour of the physicians [13]. The course consisted of mixed interactive and didactic sessions, use of multi-media, multiple exposures, needs assessment and small-group learning mentioned in reviews as effective in changing physicians' behaviour [13]–[15]. Table 1 presents the objectives, content and educational format. The participants had to study a comprehensive syllabus and received an EBM handbook; they practiced the well known EBM steps [19]: formulating questions, searching for evidence, critically appraising the literature, and applying the evidence to their case evaluations. Moreover, they could practice an EBM introductory e-lesson [20], thereby introducing an extra medium to assist the aim of changing physicians' behaviour in our intervention. Experts and teachers from the Dutch Cochrane Center, the Netherlands School of Public and Occupational Health, the Coronel Institute of Occupational Health and the Library of the Academic Medical Center were involved in the course development and teaching. Specific tools were developed, such as a model introducing key knowledge questions in the field of disability evaluation, and instructions were developed for the study subjects on how to search for evidence. The course emphasised using aggregated evidence, if possible, such as evidence-based guidelines, before using evidence from primary studies, in accordance with current common practice in EBM teaching [21]. In between course days, participants did homework assignments that served as training in the four steps of this method [19] using both case scenarios that were provided and their own cases. A logbook with all EBM steps [11], [22] was adapted for this course. The intervention group had full access to the electronic library of the Academic Medical Center, Amsterdam, thereby lowering a well-known barrier for the use of evidence in the literature [23]. High attendance at this programme, a positive factor in reviews for changing physicians' behaviour [13] was achieved both by the reward of CME points and the formal obligation that went along with this: 100% attendance was necessary to get accreditation.

**Table 1:** Characteristics of the clinically integrated post-graduate training programme in EBM for insurance physicians.

**EBM course content**Day 1: R*efresher of EBM* – general EBM knowledge; formulating answerable questions; searching for aggregated evidence.Day 2: *Systematic reviews and guidelines* – methodology, critical appraisal and searching in Medline.Day 3: *Therapy and prevention* – methodology, critical appraisal and searching for intervention studies.Day 4: *Prognosis and aetiology* – methodology, critical appraisal and searching for prognostic and aetiological studies.Day 5: *Diagnostic studies & implementation -*methodology, critical appraisal, implementation of evidence-based decision making in daily practice, personal development plans.**Objectives**For the participants in the EBM training programme:1. Know which questions are suitable for the EBM method.2. Can transform daily questions into answerable questions (with help of the PICO method).3. Can develop and execute a search strategy in different databases.4. Know how to use the concept of ‘levels of evidence’.5. Can critically appraise articles, guidelines and reviews.6. Can formulate an answer to the original question and apply it in daily practice.7. Can execute all EBM steps and can record these steps in written format, including the use of a logbook (such as a ‘CAT’).8. Are able to support colleagues with knowledge questions in practice.**Educational format**Several assignments and interactive educational formats were used:*1. Assignments for training programme preparation:* Every participant completed two assignments in advance: an interactive EBM e-course (internet) and reading the course syllabus.*2. Peer teaching:* Participants prepared a presentation about one EBM step in a group (2-3) on the course day, which was presented to the rest of the participants (12).*3. Interactive training in searching electronic databases:* Hands-on searches were practiced in Medline and in databases with aggregated knowledge such as Cochrane database, Guidelines Clearinghouse and TRIP database. A short plenary introduction was offered in advance by clinical librarians who were also tutors during these (computer) sessions.*4. Lectures:* Lectures in critical appraisal / methodology of intervention, prognostic, aetiological and diagnostic studies were given by lecturers of the Dutch Cochrane Collaboration.*5. Practical exercises:* Participants practised critical appraisal in small tutor groups (a maximum of 12 persons), building on the other educational formats. Two experienced tutors per group who were knowledgeable in EBM, epidemiology and disability assessments facilitated this process.*6. Brainstorming sessions:* Brainstorming sessions took place during interactive group discussions, allowing for the exchange of physicians' suggestions and beliefs of how to report evidence in daily practice. These sessions were organised with two experienced tutors acting as facilitators.*7. Homework assignments:* For each EBM day (days 1 – 5), a homework assignment was prepared by the physicians that followed the themes of the next EBM day Both constructed case studies and practical cases from the physician's own workplaces were used.*8. Feedback:* Feedback (e.g., on assignments or lectures, etc., and as requested by the physicians) were integrated in between course days in the educational formats 1 through 7.

In contrast, the control group had none of the above education and facilities. They knew that they would be provided with the same course one year after the start of the trial.

### Outcomes

At baseline, all participants completed a form on personal and work characteristics: age, sex, work experience, experience in specific types of disability evaluations and their experience with EBM and research. The primary outcome measurements in this study were performed three months after completing the six-month course for practical reasons (i.e., 9 months after start of the intervention). The secondary outcome measurements took place at baseline, 7 months and 12 months.

#### Primary outcomes

The primary outcome was evidence-based disability evaluation, as indicated by the frequency in use of evidence of sufficient quality in the disability evaluation reports. The quality of the evidence was measured using quality indicators [24], [25] reflecting the well-known EBM steps [19]. We defined the following quality indicators: 1) presence of evidence; 2) discernible EBM question; 3) search strategy; 4) EBM source; 5) evaluation of the quality of the evidence, and 6) actual use of evidence in underpinning of the conclusion. For each quality indicator, we developed criteria that determined whether the performance was sufficient for this indicator. Based on this judgement, a report could achieve a maximum score of 6 points. The criteria for these quality indicators were developed by the authors and were refined in 3 consensus meetings. The scores on 3 reports were compared among the researchers, and adaptations in the criteria were made until sufficient agreement was reached. The quality indicators with their criteria are listed in Appendix S2.

The participating physicians knew in advance when the reports were demanded and provided all disability evaluation reports on all their patients during the first two weeks in February 2010. We needed their help in collecting the reports because it was not feasible to just extract the reports from an administrative system.To measure the quality of the disability report, we applied the quality indicators to all disability evaluation reports from all participating physicians that we collected during these two weeks.

We asked the participating physicians to note for each report if they had used evidence as part of their professional judgement. If evidence was used, these reports were selected, and two research assistants who were trained in research methods and in the scoring of the indicators independently scored the quality indicators of these reports. When scores differed, discussion ensued until consensus was reached.

Among the reports in which the physicians indicated they had not used evidence, two of the authors (SK and RK) independently checked a sample of 50 randomly selected reports and verified that there was no use of evidence. All reports for which the physicians indicated that they had not used evidence were scored zero on ‘quality of disability report’.

Thus, a report could obtain a score ranging between 0 and 6 for the ‘quality of disability report’. An a priori cut-off of 3 points was rated as sufficient ‘quality of disability report’. Finally, our primary outcome was measured by calculating the percentage of reports in each group (intervention vs. control) in which quality was assessed as sufficient. And more specifically we were interested in the absolute difference in proportions between these two groups as the potential reflection of the effect of this large EBM intervention.

Before the assessments, the personal details of physician and patient were removed from each report.

#### Secondary outcome measures

Knowledge and skills in EBM were assessed by the validated Fresno test [26], [27]. We adapted the test so that the scenarios were applicable to the context of disability evaluation. We maintained the standardised grading system of the Fresno Test (scores ranged from 0–212).

Intention to change behaviour was assessed with 22 statements that could be answered using a 5-point Likert scale [11], [12]. The statements referred to five constructs within the attitude-social influence-(self)-efficacy (ASE) model [28]: attitude towards EBM, influence of social context on EBM, self-efficacy in performing EBM, intention to use evidence and the self-reported use of evidence. We calculated a mean score for each of the constructs (scores ranged from 0 to 5).

We hypothesised that the physicians' appreciation of their own profession would become more favourable as a result of the training in EBM. We measured this attitude with 10 statements on a visual analogue scale (VAS) ranging 0–10 points, with the anchors as ‘fully disagree’ (0 points) and ‘fully agree’ (10 points). Sum scores were constructed.

In line with earlier research, we defined professional performance as the self-reported practice of keeping up with and using evidence-based knowledge in daily practice [29], summarised in one sum score (0–27 points). The scale included questions to determine the amount of time spent on keeping current with research and the extent of use of the Internet and literature databases.

#### Process variables

At the end of each day of our intervention, different aspects of the education programme were evaluated with a questionnaire.

### Statistical analysis

For our primary binary outcome measure, logistic multilevel analysis was used to analyse if there was a difference in the use of evidence of sufficient quality in the disability evaluation reports between the intervention and control groups. We distinguished several levels of data: the first level was the disability evaluation report, the second level was the writing of the disability report by the physician, and the third level was the case-based learning group representing several physicians writing disability evaluation reports. This clustering of our data was adjusted using a generalized linear mixed model (GLMM) analysis for binary outcomes in SPSS 19.0 and was performed in close collaboration with a statistician. In this model, we used intervention or control group membership as a fixed effect. Membership of case-based learning group was treated as random effect. Scores of patient disability evaluation reports from one physician were entered as repeated measurements at a single moment. The variance-covariance matrix, modelling the correlation structures of the measurements, with the optimal score on Akaike's information criteria (AIC), was chosen [30]. We calculated intra-cluster correlations using a proportion of variance interpretation, and we used Swiger's formula to calculate the concomitant confidence intervals [31].

For all secondary continuous outcome measures, a two-level linear regression analysis with a similar Linear Mixed Model (LMM) in SPSS 19.0 was based on repeated measurements in time (level 1), with clustering at level 2 for the case-based learning groups. In this model, we focused on the group x time interaction as a fixed effect, as this would demonstrate a learning effect. All of the outcome measures were analysed according to the original randomisation scheme as a so-called intention-to-treat-analysis. The intra-cluster correlation was calculated using an ANOVA procedure, and Swiger's formula was used to calculate the concomitant confidence intervals [31].

We performed a missing-value analysis by comparing participants who did not send in reports to those who did using baseline prognostic variables, such as previous experience with EBM, experience with critical appraisal and experience with research. Moreover, a sensitivity analysis was performed using best- and worst-case scenarios. In the best-case scenario, we substituted all missing values with the mean score in the highest quartile of the physicians who did send in reports, whereas in the worst-case scenario, we supposed that the missing reports all scored ‘0’.

We used the items of the CONSORT statement for improving the quality of reporting cluster-randomised trials [32].

## Results

Recruitment took place between January and April 2009. Seventy-eight teams consisting of approximately 700 physicians existed in the Netherlands. However, 14 teams had a group leader that led more than one group. Because of the risk of contamination, this was not allowed; therefore, only sixty-four teams consisting of approximately 574 physicians were potentially eligible for this trial. Five teams did not respond to our invitation for unknown reasons. Thus, fifty-nine teams consisting of 147 physicians were eligible for the trial and were invited to participate. Five teams (15 physicians) declined participation because of organisational barriers unrelated to the study.

After cluster randomisation, 27 teams consisting of 67 physicians were assigned to the intervention ‘EBM’ group, and another 27 teams consisting of 65 physicians were assigned to the waiting list control group, yielding a total of 132 participating physicians. The first day of the EBM course started in May 2009.

Four physicians assigned to the intervention group declined participation before the start of the EBM course because they felt they were too busy, and another three physicians had to discontinue the course during the intervention period due to illness or unknown reasons. In the control group, at baseline, one physician was excluded due to illness, one was unwilling and the third was not employed anymore.

**Figure 1:**
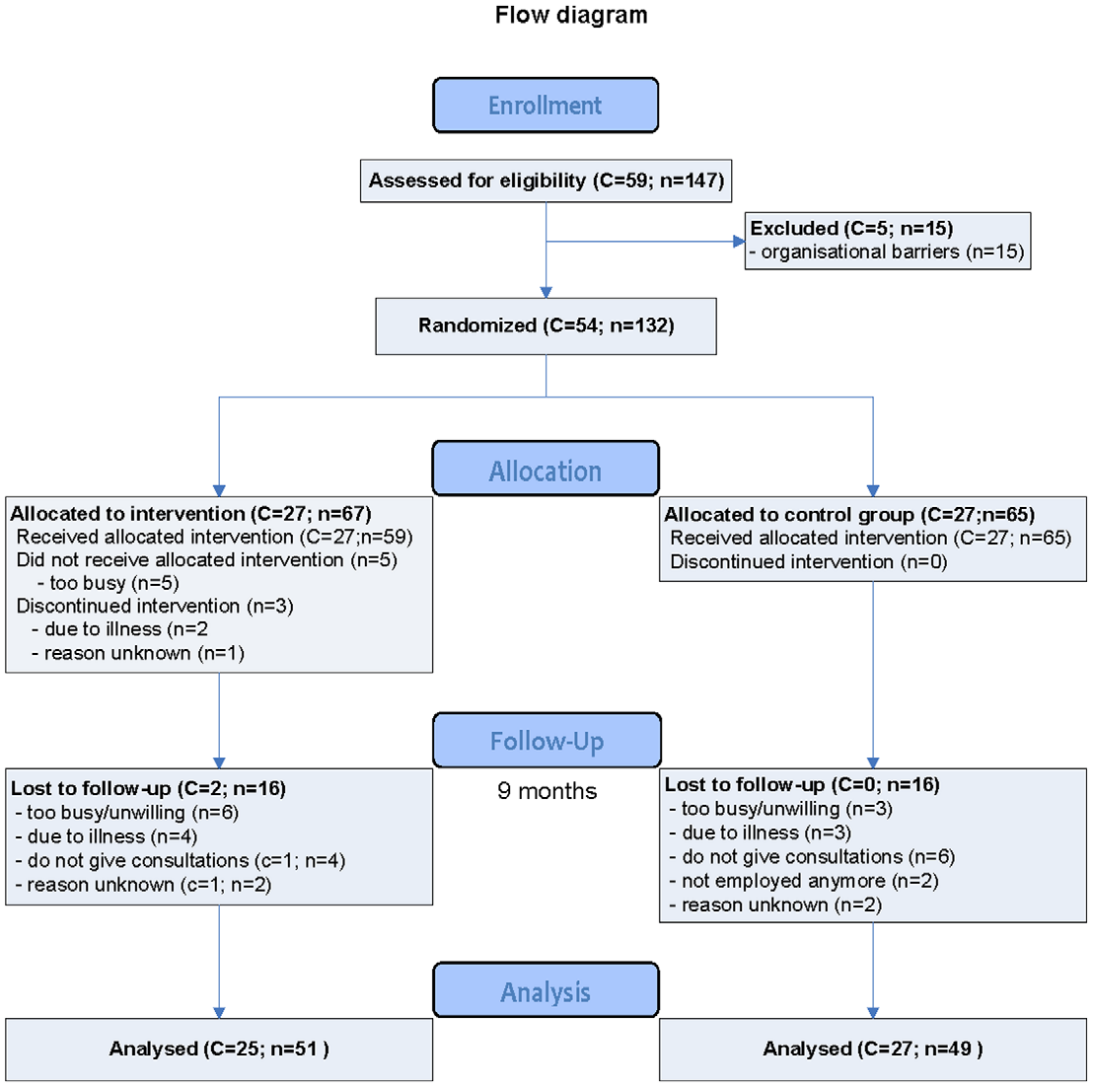
Flow of clusters (c) and physicians (n) through the trial for primary outcome analysis.

Loss to follow-up varied depending on the outcome measure used. More physicians completed the secondary outcome measurements (n = 125 at baseline, n = 117 at 7 months and n = 111 at 12 months) compared to the primary outcome measurements (n = 100 at 9 months). The overall number and reasons for loss to follow-up for the primary outcome measurements were similar (see Figure 1). The main reason for the lower number of physicians available for the primary outcome analyses was that some physicians (n = 12) did not consult during the evaluation period, which made it impossible for them to send in any reports. Another reason was that physicians were too busy (n = 9). In total, 32 physicians, equally divided among both groups, did not provide disability evaluation reports, resulting in data from 100 physicians being available for the intention to treat analyses of the primary outcome ‘evidence-based disability evaluation’ (see Figure 1).

Table 2 shows the baseline characteristics of the groups and the physicians. The number of clusters (N = 27) and mean cluster size (a mean of 2.44 in the control group and 2.48 in the experimental group) were comparable. We did not observe relevant differences in socio-demographic characteristics or in baseline outcome measurements between groups.

**Table 2:** Baseline characteristics of case-based learning groups (clusters) and physicians in intervention and control groups.

Characteristics of case-based learning groups (clusters)	Intervention	Control
Clusters, N Mean cluster size	27 2,5	27 2,4
**Characteristics of physicians**		
N* Age, mean (SD) Male, N (%) Experience as MD in years, mean (SD) Postgraduate qualification, N (%) Work experience as insurance physician in years, mean (SD) Hours work on weekly basis, mean (SD) Previous experience with EBM, N (%) Previous experience with critical appraisal, N (%) Previous experience with research, N (%)	62 49,7 (7,1) 41 (66,1) 21,6 (6,8) 59 (90,8) 16,4 (6,7) 34,6 (6,9) 45 (72,6) 17 (27,9) 51 (83,6)	63 48,7 (6,3) 35 (55,6) 21,5 (6,7) 59 (93,7) 16,0 (7,5) 33,0 (6,7) 44 (69,8) 15 (23,8) 53 (84,1)

* For the various parameters numbers varied due to missing values.

### Primary outcome

In the intervention group, 16.7% of the reports indicated that physicians performed evidence-based disability evaluations (a ‘quality of disability report’ score of three or more), compared to 7.0% of the reports of the physicians in the control group, a statistically significant absolute difference of 9.7% (95% CI of 3.5 to 15.9) (see also Table 3) with the fixed effect for intervention statistically significant at p = 0.002. The intra-cluster correlation (ICC) for disability evaluation reports per physician was 0.5 (95% CI of 0.42 to 0.58), and the ICC for physicians per case-based learning group was negligible.

**Table 3:** Primary and secondary outcome measures: results for intervention (I) and control (C) group from baseline up to 12 months, and differences between groups.

Primary outcome: measured at 9 months	Intervention, % (sd)	Control, % (sd)	Mean difference (95% CI) Intervention – Control	Mixed model analyses: Fixed effect ‘intervention’; ICC physicians (95%CI)
Evidence-based disability evaluations across physicians (n = 100 physicians, n = 1680 disability evaluations)	16.7 (19.0)	7.0 (11.2)	9.7 (3.5; 15.9)*	F = 9.2;df = 1678;p = 0.002 ICC = 0.5 (0.42;0.58)
**Secondary outcomes:** measured at baseline (n = 125 physicians), 7 months (n = 117), 12 months (n = 111)	Intervention, mean (sd)	Control, mean (sd)	Mean difference (95% CI) I – C	Mixed model analyses: Fixed effect ‘intervention x time’; ICC groups (95% CI)
Knowledge/skills in EBM (0-212) 0m	93.7 (25.9)	88.7 (33.8)	5.0 (−5.6;15.7)	F = 18.8; df = 110.4; p = 0.000 ICC = −0.043 (−0.24;0.15)
7m	128.2 (22.6)	95.2 (30.4)	33.0 (23.2;42.9)*	
12m	121.7 (25.1)	92.6(32.9)	29.2 (18.2; 40.2)*	
Attitude towards EBM(1-5) 0m	4.0 (0.3)	3.9 (0.5)	0.06 (−0.08; 0.2)	F = 0.8; df = 115.5; p = 0.5 ICC = 0.028 (−0.18;0.23)
7m	4.0 (0.4)	3.9 (0.5)	0.1 (−0.04; 0.3)	
12m	4.0 (0.4)	3.8 (0.5)	0.2 (−0.01; 0.3)	
Influence Social context on EBM (1–5) 0m	2.9 (0.5)	2.9 (0.6)	0.05 (−0.1;0.2)	F = 0.2; df = 117.4; p = 0.8 ICC = 0.001 (−0.20;0.20)
7m	3.0 (0.5)	2.9 (0.6)	0.05 (−0.1;0.2)	
12m	3.0 (0.5)	2.9 (0.5)	0.1 (−0.1;0.3)	
Self-efficacy in performing EBM(1–5) 0m	2.6 (0.5)	2.8 (0.5)	−0.1 (−0.3;0.04)	F = 33.1; df = 114.5; p = 0.000 ICC = 0.003 (−0.20;0.21)
7m	3.2 (0.5)	2.7 (0.6)	0.5 (0.3;0.7)*	
12m	3.1 (0.5)	2.6 (0.5)	0.5 (0.3;0.7)*	
Intention to EBM behavior (1–5) 0m	3.9 (0.4)	3.8 (0.5)	0.03 (−0.1;0.2)	F = 0.5; df = 115.8; p = 0.6 ICC = −0.091 (−0.28;0.10)
7m	3.9 (0.4)	3.8 (0.5)	0.1 (−0.07;0.3)	
12m	3.7 (0.5)	3.6 (0.5)	0.08 (−0.09;0.3)	
Self-reported use of evidence (1–5) 0m	3.2 (0.6)	3.2 (0.6)	−0.05 (−0.3;0.2)	F = 4.3; df = 115.7; p = 0.02 ICC = 0.15 (−0.056;0.36)
7m	3.5 (0.5)	3.3 (0.6)	0.2 (−0.06;0.4)	
12m	3.6 (0.6)	3.3 (0.7)	0.3 (0.02; 0.5)*	
Appreciation own profession (0–100) 0m	68.3 (9.5)	66.0 (11.9)	2.4 (−1.5;6.2)	F = 3.5; df = 112.6; p = 0.04 ICC = −0.019 (−0.22;0.18)
7m	70.8 (10.2)	69.2 (12.1)	1.6 (-2.5;5.7)	
12m	70.5 (8.9)	64.0 (13.2)	6.4 (2.2;10.7)*	
Professional performance (0-27) 0m	20.0 (1.5)	20.0 (3.1)	−0.09 (−0.9;0.8)	F = 1.0; df = 85.7; p = 0.4 ICC = −0.098 (−0.29;0.093)
7m	21.5 (2.1)	20.9 (2.2)	0.7 (−0.09;1.5)	
12m	21.6 (2.2)	21.1 (2.2)	0.5 (−0.3;1.3)	

* p<0.05 with t-test and mixed model analysis.

### Secondary outcomes

Knowledge and skills in evidence-based medicine, as measured by the Fresno test, improved more over time in the intervention group than in the control group (Mixed model analyses, p = 0.000) (Table 2). At 7 months, the physicians in the intervention group had a mean Fresno score of 128.2 (SD 22.6) compared to 95.2 (SD 30.4) in the control group, a mean difference of 33.0 (95% CI 23.2 to 42.9). This difference declined only slightly at 12 months to a mean difference of 29.2 (95% CI 18.2 to 40.2) (Table 3).

The physicians' attitude towards EBM was similar in both groups after the intervention at 7 months (4.0 vs. 3.9) and at 12 months (4.0 vs. 3.8). The perceived influence of the social context was also similar (3.0 vs. 2.9 at 7 months and 3.1 vs. 2.9 at 12 months) and showed no differences in improvement between groups.

The physicians in the intervention group were more confident in using EBM compared to the control group (self-efficacy mean 3.2 vs. 2.7 at 7 months and 3.1 vs. 2.6 at 12 months), but intention to behaviour was not influenced (Table 3). The self-reported use of evidence in daily practice was higher in the intervention groups compared to the control group at 12 months (3.6 vs. 3.3). The ‘appreciation of own profession’ did not change directly after the intervention but was higher in the intervention group at 12 months follow-up (70.5 vs. 64.0). Professional performance was not significantly different between groups.

### Sensitivity analysis

We observed no differences in baseline prognostic variables such as previous experience with EBM, experience with critical appraisal or experience with research between physicians who did not send in reports (n = 32) and those who did (n = 100). In the best-case scenario in our sensitivity analysis, the estimated mean difference between the control and intervention group was somewhat reduced to 6.9% (95% CI of 1.0 to 13.0). In the worst-case scenario, the estimated mean difference became 6.5% (95% CI of 2 to 11%).

## Discussion

A clinically integrated post-graduate training programme in EBM results in more evidence-based disability evaluation. This outcome was accomplished by a concurrent increase in knowledge and skills in EBM and a higher rating of self-efficacy.

### Strengths and limitations of the study

A strength of our study is that we applied the EBM model in a non-hospital setting and evaluated it in a randomised controlled trial that used a large sample of physicians and disability evaluation reports.

Furthermore we measured a very concrete behavior of physicians: whether they used evidence to support their decisions or not. We believe that our outcome is therefore important for health care and patients. Work and work ability are important aspects of quality of life and, thus, their assessment by physicians is important as well. Physicians all over the world make judgments about work ability and work capacity of their patients [7]. Where this can be underpinned with evidence from scientific research, it will improve the quality of care.

In our intervention, we used components known to be successful in influencing physicians' behaviour [13]–[15]. The participating physicians were enthusiastic about the EBM course and rated it at a mean of 8.1 on a scale of 0–10. An increase from 7.0% to 16.7% in evidence-based disability evaluation in favour of the intervention group may not seem impressive; however, one has to take into account that this is based on the proportion of all disability evaluation reports. The majority of these reports are routine cases, and the insurance physicians do not see a need to use evidence to underpin their decisions [12]. Thus, the proportion of reports in which sufficient evidence is used and where it was needed is much higher, but we felt that it was too difficult to develop valid criteria to assess this aspect.

In terms of the appreciation of the educational programme, we believe we did exceptionally well; the participants were very enthusiastic. Compared to other experiences, the satisfaction ratings were very high; given the considerable travelling time that many participants had, the attendance rate was high.

This enthusiasm can also be a drawback in that these (insurance) physicians were probably the more enthusiastic colleagues for using EBM (pioneers). Therefore the generalization of this results to all (insurance) physicians working for the Dutch National Institute of Benefit Schemes, should be taken with caution. However we did include 1 out of 7 (insurance) physicians working for this institute, which is considerable enough to assume that not everyone was a pioneer in advance. Another limitation of our study is that no validated relevant general outcome measures were available; thus, our primary outcome had to be constructed for this study. However, we followed general guidelines for constructing performance indicators and defined an a priori cut-off for determination of sufficient quality as recommended by Shaneyfelt for evaluating EB training [24]–[26]. Therefore, we believe that the outcome is a valid and relevant indicator of physicians' behaviour in using evidence for disability evaluations.

We also had a considerable loss to follow-up (24%) for our primary outcome measure. In educational research, it is not easy to randomise and retain participants in the intervention and control groups. Participants know to which group they belong, and it is often more convenient for them to change groups. To prevent losing the benefits of randomisation, we strictly adhered to the allocation to the intervention and control groups. That is one reason why we lost participants. However, our sensitivity analysis showed that it is unlikely that this has influenced our main findings.

Even though the intervention group increased considerably in knowledge, the mean score on the Fresno test was approximately half of the maximum score. However, with an effect size of 1.10 (MD/SD baseline), our course compares favourably with the results of other EBM courses that on average yielded an effect size of 0.44 [33]. Ramos reports a mean Fresno score of 95.6 for novices and of 147.5 for experts. This means that our course brought the participants on average more than half-way from novice to expert-level [27]. Nevertheless, many EBM concepts remain difficult to understand for the participants, such as the difference between relative risk and probability in prognosis or the difference between odds ratios and relative risks when the prevalence of the disease is high. It also takes time for participants to feel that they are confident in making a judgement of the quality of an article. This means that a continued effort in learning about EBM is needed but we need also better information management tools to make EMB more feasible [34].

As a limitation we could further add that we are not able to differentiate which educational element was effective in effectuating the improved use of evidence in the disability reports.

Theoretically, one could argue that the attention resulting from the intervention and not the intervention itself would have led to the effect. Even though this might be the case in drug studies, we believe that educational interventions are different and that just giving attention to persons will not increase their knowledge and skills. This does not mean that asking for all of their reports in advance, is not having an effect. We strongly believe, that if physicians are capable to use EBM, asking for their reports, leads to improved use of evidence. Moreover we found a parallel improved score change in the adapted Fresno questionnaire which further supports our strong belief that improved knowledge and skills in EBM are responsible for the improved use of evidence in disability reports.

### Comparison with other studies

As in other studies in which the educational intervention was integrated in routine practice, our intervention significantly improved knowledge [9]. Other studies report also changes in behaviour but most of them are based on self-reported behaviour which is notorious for being misjudged and the participants are residents and not practising physicians [9], [26]. We know of only three other randomised studies that measured behaviour change among practising physicians as a result of EBM training integrated in routine practice. One study, among public health physicians, objectively measured behaviour change but did not find an effect on behaviour, which was possibly due to extreme loss to follow-up [5]. Another randomised study among primary care physicians did not find a significant difference in a variety of clinical behaviours such as drug prescription, test ordering or clinical examinations between an EBM trained and a control group [10]. This might have been due to the baseline rate of clinical behaviour being low, as argued by Glasziou and thus the study is underpowered for these outcomes [35]. A third study also showed that a training programme in EBM changed occupational health physicians' behaviour and led to more frequent high-quality advice regarding return to work interventions and prognoses [11]. However, the outcome measure in their study differed from our study in that the authors only measured the quality of a limited, self-selected number of cases, whereas we sampled all reports in a certain period of time. In both their and our study, the EBM course was practice-based, with real cases, experienced teachers and opportunities to interact; this may have contributed to the increase in self-efficacy, an important step for implementing new practices [5].

The lack of an increase in positive attitude towards EBM in this study is in line with our earlier finding in a one-day workshop [12]. We believe that this is due to a ceiling effect because insurance physicians are already very motivated to use evidence in their reports. They feel that it prevents appeals against their decisions and makes their cases stronger compared to the opinions of other experts such as medical specialists in hospitals. We thought that this would also lead to a better appreciation of their profession, but the changes were small and only significant at 12 months follow-up.

### Implications for practice and further research

We showed that a clinically integrated post-graduate training programme in EBM successfully improved knowledge, skills and self-efficacy of physicians and their use of evidence in a non-hospital based medical specialty. The educational components of our intervention are also applicable to physicians in other specialties and will enhance their use of evidence for clinical decision-making. In our study, we believe it was important that the professional context of the intervention was very supportive in the sense that searches in databases, using and applying guidelines and other forms of evidence are considered standard practice and are encouraged by colleagues and management. Thus, in helping doctors make better decisions, we believe that the context in which physicians work has to be supportive. In general, a good knowledge infrastructure, especially the availability of online full-text guidelines, reviews and articles, is a prerequisite [36]. As with all education, physicians should keep their knowledge up to date and practice their skills. For this reason all participants of this trial still have EBM refreshing days every year and are starting with EBM groups to ensure regularly practicing.

## Supporting Information

Appendix S1 Functional abilities list.
(PDF)

Appendix S2 List of quality indicators with criteria for the primary outcome ‘evidence based disability evaluation’.
(PDF)
