# Supplementary material for: A Clinically Integrated Post-Graduate Training Programme in Evidence-Based Medicine versus ‘No Intervention’ for Improving Disability Evaluations: A Cluster Randomised Clinical Trial
Source: PLoS One. 2013 Mar 1;8(3):e57256. doi: 10.1371/journal.pone.0057256 (PMC3585805; doi:10.1371/journal.pone.0057256)
Supplement: Appendix S1 — Functional abilities list. (PDF) [file pone.0057256.s001.pdf]

Appendix S1: FUNCTIONAL ABILITIES LIST

---

*This list is an overview of an individual's **general** functional abilities during a full working day (minimum of 8 hours). **Restrictions** to these abilities with regard to **normal values** are given in a separate list if considered symptomatic of an illness, incapacity or accident in the opinion of the insurance company doctor. The standard functional levels required in daily life have been taken as the normal values. Unless expressly stated otherwise, incidental **peak demands** above the given functional levels are also possible.*

*This list should only be applied if accompanied by a **medical insurance physician's report** that, based on an analysis of the problem, evaluates, motivates and describes the correlation of functional abilities and limitations.*

Name:...

National insurance number:..

....sex***: m/f***

Diagnosis code: ....

Last/current work:...

(hours per week:**...................**)

Resumed work: ....

(hours per week:**..................**)

Conclusion:

O The client has long-term capacities for work
O The client does not have long-term capacities for work

Explanation:

O The client is capable of fully functioning in his own job/function
O The client is capable of functioning normally (see headings)
O The client's normal functioning is impaired (see headings)
O Other, see report by the insurance company doctor

O The client's personal and/or social functioning is extremely limited (see headings I, II)
O The client has been admitted to a hospital or institution recognised by the Exceptional Medical Expenses Compensation Act (AWBZ)
O The client is bedbound (for most of the day, long-term)
O The client is highly dependent as regards performing daily living activities (*ADL*)
O The client has highly variable functional abilities/loss of functional abilities < 3 months - 1 year

---

Date:.....................................**Insurance physician :**..

HEADING I: PERSONAL FUNCTIONING

1. Focusing attention

0 Normal, can concentrate on an information source (book, documentary on TV or radio) for at least half an hour)
1 Limited, cannot concentrate on an information source (newspaper, current affairs programme on radio or TV) for more than half an hour
2 Very limited, cannot concentrate on an information source (advertising brochure, TV or radio advert) for longer than 5 minutes

2. Dividing attention

0 normal, can concentrate for at least half on a number of information sources (can manage driving or cycling in busy traffic)
1 limited, cannot concentrate for at least half on a number of information sources (can manage driving or cycling in busy traffic)
2 Very limited, cannot concentrate for longer than 5 minutes on a number of information sources (crossing a busy street alone)

3. Memory

0 normal, can generally remember relevant things promptly, without resorting to unusual aids
1 limited, must frequently write things down as a memory aid to safeguard the continuity of his actions
2 very limited, constantly unable to remember essential everyday things (time, place, person, subject), and cannot compensate with memory aids

4. Insight into own abilities

0 normal, mostly estimates own abilities and limitations reasonably accurately
1 limited, generally highly overestimates own abilities
2 limited, generally highly overestimates own limitations

5. Effective action (task implementation)
(coordinated action, gears own activities to realising a goal)

0 normal, no specific limitations to his effective action. The routine of daily life (getting up on time, washing, dressing, preparing breakfast, breakfasting, locking up the house and arriving at appointments on time)
1 limited, does not commence activities on time in order to realise set goal
2 limited, does not conduct the necessary activities in a logical order
3 limited, does not check the course of the activities
4 limited, does not end the activities once set goal is reached or cannot be reached
5 otherwise limited in taking effective action, i.e. ....

6. Independent action (carrying out tasks autonomously)

0 normal, no specific limitations to independent action in daily life
1 limited, does not generally initiate action
2 limited, does not generally set himself goals
3 limited, does not generally think of variations on a task independently
4 limited, generally does not generally take an independent decision on the best approach to take
5 limited, does not generally realise when the decided approach falls short
6 limited, in those instances, does not generally take an independent decision to follow an alternative line of action or set a different goal
7 limited, does not generally continue, under own initiative, until goal is accomplished
8 limited, does not call on others promptly for help when the situation demands
9 otherwise limited in independent action, i.e. ....

7. Action tempo

0 normal, there are no specific limitations to the action tempo in daily life
1 limited, the action tempo is considerably slower

8. Other limitations to personal functioning

0 normal, no other specific limitations to personal functioning in daily life
1 limited, other specific limitations, i.e. ....

9. Specific conditions for personal functioning in a work situation
(is work functioning dependent on specific conditions because of the said limitations or the client's compensatory behaviour?)

0 no, there are no specific conditions for personal work functioning
1 yes, the client has been advised to follow a fully pre-structured work schedule: concrete, one-sided assignments (what, when, how long; one task per assignment) and to follow prescribed implementation orders (how)
2 yes, the client has been advised to follow fixed, familiar working methods (routine-dependent)
3 yes, the client has been advised to perform work under immediate supervision (consistent feedback) and/or to work under intensive supervision
4 yes, the client has been advised to work in a situation in which he is not distracted by the activities of others
5 yes, the client has been advised to work in a predictable working situation, cannot respond flexibly to highly varied situations in which in work s performed and/or varied work content
6 yes, the client has been advised to work in a work situation not susceptible to constant interruptions and disturbance
7 yes, the client has been advised to work in a work situation not susceptible to constant deadlines or production peaks
8 yes, the client has been advised to work in a work situation in which a high action tempo is not required
9 yes, the client has been advised to work in a work situation in which there is no increased personal risk
10 yes, there are other specific conditions, i.e. ....

Explanation: *see medical insurance physician's report*

HEADING II: SOCIAL FUNCTIONING

1. Vision

0 normal, no specific limitation in daily functioning
1 limited, i.e. ....

2. Hearing

0 normal, no specific limitation in daily functioning
1 limited, i.e. ....

3. Speech

0 normal, no specific limitation in daily functioning
1 limited, i.e. ....

4. Writing

0 normal, no specific limitation in daily functioning
1 limited, i.e. ....

5. Reading

0 normal, no specific limitation in daily functioning
1 limited, i.e. ....

6. Dealing with the emotional problems of others

0 normal, can generally empathise with the problems of others but can also distance himself in terms of behaviour and experience
1 limited, generally becomes involves in the problems of others; nevertheless, can distance himself sufficiently in terms of behaviour although not experience
2 very limited, generally identifies with the problems of others and cannot distance himself in terms of either behaviour or experience

7. Expressing personal feelings

0 normal, can generally express personal feelings in a way acceptable to others, both verbally and behaviourally
1 limited, confuses others with unpredictable or unconventional ways of expressing feelings
2 very limited, is generally incapable of expressing feelings (blocks himself) or expresses them in an uncontrolled way regardless of the feelings of others

8. Dealing with conflicts

0 normal, can directly deal with conflicts with aggressive or unreasonable people
1 limited, can only deal with conflicts with aggressive or unreasonable people by phone or in writing
2 very limited, cannot generally deal with conflicts

9. Working with others

0 normal, can jointly carry out a task with others (teamwork)
1 limited, can work with others but only with a task of his own, clearly defined beforehand
2 very limited, as a rule is unable to work with others

10. Transportation

0 normal, can drive or cycle or use public transport on his own
1 limited, is reliant on others for transportation

11. Other limitations to social functioning

0 normal, no other specific limitations to social functioning in daily life
1 limited, other specific limitations, i.e. ....

12. Specific conditions for social functioning at work
(is social functioning at work dependent on specific conditions because of the said limitations or the client's compensatory behaviour?)

0 no, there are no specific conditions for social functioning at work
1 yes, the client has been advised to work in a situation demanding no direct contact with clients (some occupations in the service sector)
2 yes, the client has been advised to work in a situation where little or no direct contact with patients or those needing help is required (some occupations in the health care sector)
3 yes, the client has been advised to work in a situation in which, if necessary, he can fall back on immediate colleagues or managers (no solitary job)
4 yes, the client has been advised to work in a situation which generally does not require direct contact with colleagues
5 yes, the client has been advised to work in a situation involving no managerial aspects
6 yes, there are other specific conditions, i.e. ....

Explanation: *see medical insurance physician's report*

HEADING III: ADJUSTING TO PHYSICAL ENVIRONMENT

1. Heat

0 normal, no specific limitations
1 limited, i.e. ....

2. Cold

0 normal, no specific limitations
1 limited, i.e. ....

3. Draught

0 normal, no specific limitations
1 limited, i.e. ....

4. Skin contact

0 normal, no specific limitations
1 limited, i.e. ....

5. Protective measures

0 normal, no specific limitations
1 limited, i.e. ....

6. Dust, smoke, gases and fumes

0 normal, no specific limitations
1 limited, i.e. ....

7. Noise nuisance

0 normal, no specific limitations
1 limited, i.e. ....

8. Vibration

0 normal, no specific limitations
1 limited, i.e. ....

9. Other limitations to physical adjustment abilities

0 normal, no other specific limitations to physical adjustment abilities
1 allergies, i.e. ....
2 increased susceptibility to infections, i.e. ....
3 weakened skin barrier, i.e. ....
4 other limitations, i.e. ....

10. Specific conditions for adapting to the physical working environment
(is adjustment to the working environment dependent on specific conditions because of the said limitations or the client's compensatory behaviour?)

0 no, there are no specific conditions for adapting to the physical working environment
1 yes, there are specific conditions for adapting to the physical working environment, i.e. ....

Explanation: *see medical insurance physician's report*

HEADING IV: DYNAMIC MOVEMENT

1. Dominance

0 not applicable
1 right
2 left

2. Localisation limitations

0 neither right nor left
1 right
2 left
3 both sides

3. Use of hand and fingers

0 normal, no specific limitations when using hands and fingers in daily life
1 limited, can hardly perform a ball grip, if at all
2 limited, can hardly perform a pen grip, if at all
3 limited, can perform a pincer grip, if at all
4 limited, can perform a key grip, if at all
5 limited, can perform a cylinder grip, if at all
6 limited, can use hand/fingers to squeeze or grip , if at all
7 limited, is hardly able to perform fine motor hand/finger movements
8 limited, is not able to perform repetitive hand/finger movements, if at all

4. Touch

0 normal, no specific limitations in daily life
1 limited, i.e. ....

5. Using a keyboard and mouse

0 normal, can perform all required movements
1 limited, i.e. ....

6. Working with a keyboard and mouse

0 normal, if required can use a keyboard and mouse most of the working day (professional word-processing, programming, CAD/CAM work, electronic sales)
1 slightly limited, if required can use a keyboard and mouse half the working day (roughly 4 hours) (policy worker)
2 limited, if required can use a keyboard and most for a small part of the working day (roughly 1 hour) (to send email)
3 very limited, can use a keyboard and mouse less than thirty minutes a working day

7. **Twisting movement – hand and arm**

0 normal, no specific limitations in daily life
1 limited, i.e. ....

8. **Stretching arm**

0 normal, can stretch arms (serve coffee)
1 slightly limited, can stretch arm slightly (shoulder-hand distance = 50-60 cm)
2 limited, can stretch arm slightly (shoulder-hand distance = less than 50 cm)

9. **Can stretch arm frequently during work (roughly 20 times a minute)**

0 normal, if required can stretch frequently during each hour of the working day (cashier work in wholesale company, packaging work)
1 slightly limited, if required can stretch frequently for roughly 4 hours of the working day
2 limited, if required can stretch frequently roughly one hour per working day
3 very limited, cannot stretch frequently during one hour of the working day

10. **Bending**

0 normal, can bend roughly 90 degrees (pick up a piece of paper from the ground)
1 limited, can bend roughly 60 degrees (pick up a bag from the ground)
2 very limited, can bend roughly 45 degrees (pick up crumbs from a chair)

11. **Frequent bending during work (roughly ten times per minute)**

0 normal, if required, can bend frequently during each hour of the working day
1 slightly limited, if required, can bend frequently roughly 4 hours per working day
2 limited, if required, can bend frequently one hour per working day
3 very limited, cannot bend frequently one hour per working day

12. **Turning/twisting**

0 normal, can turn torso at least 45 degrees (look behind while cycling, reach into the back seat of the car to get a bag while sitting in the front)
1 limited, i.e. ....

13. **Pushing/pulling**

0 normal, can push or pull roughly 15 kgf (remove a stubborn cork from a wine bottle)
1 limited, can push or pull roughly 10 kgf (full rubbish container)
2 very limited, can push or pull roughly 5 kgf (open door with door-closer)

14. **Carrying/lifting**

0 normal, can carry roughly 15 kg (toddler)
1 slightly limited, can carry roughly 10 kg (infant)
2 limited, can carry roughly 5 kg (bag of potatoes)
3 very limited, can lift roughly 1 kg (litre container of milk)

15. Frequently managing light objects at work (roughly 10 times per hour)

0 normal, if required can manage objects weighing around 1kg frequently during every hour of the working day (order book)
1 slightly limited, if required, can manage objects of 1 kg for roughly 4 hours per working day
2 limited, if required, can manage objects of around 1 kg for roughly one hour per working day
3 very limited, cannot manage objects of around 1 kg for one hour per working day

16. Frequently managing heavy loads at work (roughly 10 times per hour)

0 normal, if required, can frequently manage loads of roughly 15 kg for one hour per working day
1 limited, cannot frequently manage loads of roughly 15 kg during one hour per working day

17. Head movements

0 normal, can move head without hindrance
1 limited, can move head to a limited extent
2 very limited, can barely turn head to the side if at all
3 very limited, can barely move head up and down if at all

18. Walking

0 normal, can walk for roughly one consecutive hour (a walk)
1 slightly limited, can walk for roughly 15-30 consecutive minutes (a stroll)
2 limited, walk for roughly 5-15 consecutive minutes (to the letterbox)
3 very limited, can walk for less than 5 consecutive minutes (indoors)

19. Walking while at work

0 normal, if required, can spend most of the working day walking (postal worker)
1 slightly limited, if required can walk half the working day (roughly 4 hours)
2 limited, if required, can walk a limited part of the day (roughly 1 hour)
3 very limited, can walk for less than half an hour per working day

20. Stairclimbing

0 normal, can walk at least 2 flights of stairs up and down in one go (2 floors of a house)
1 slightly limited, can walk at least up and down a flight of stairs in one go (1 floor of a house)
2 limited, can walk at least down stairs in one go (1 floor of a house)
3 very limited, can only walk on or off the curb in one go

21. Climbing

0 normal, can at least climb up and down a ladder (1 floor)
1 slightly limited, can at least climb up and down a household stepladder
2 limited, can at least get up and down from a stool (50 cm, elephant foot)
3 very limited, cannot climb up and down

22. Kneeling or squatting

0 normal, can touch the ground with hands when kneeling or squatting (picking up a coin)
1 limited, can barely touch the ground with hands when kneeling or squatting, if at all

23. Other limitations to dynamic movement

0 normal, no other specific limitations to dynamic movement in daily life
1 other specific limitations, i.e. ....

24. Specific conditions for dynamic movement at work
(is dynamic movement at work dependent on specific conditions because of the said limitations or the client's compensatory behaviour?)
0 no, there are no specific conditions for dynamic movement at work
1 yes, there are specific conditions for dynamic movement at work, i.e. ....

Explanation: *see medical insurance physician's report*

HEADING V: STATIC MOVEMENTS

1. Sitting

0 normal, can sit for roughly 2 consecutive hours (car journey)
1 slightly limited, can sit for roughly one consecutive hour (film)
2 limited, can sit for roughly 30 consecutive minutes (meal)
3 very limited, can sit for less than 15 consecutive minutes (TV news)

2. Sitting at work

0 normal, if required, can sit for almost the whole working day (assembly work, cashier work, administrative work)
1 slightly limited, if required can sit for most of the working day (6-8 hours)
2 limited, if required can sit for half the working day (roughly 4 hours)
3 very limited, can sit for less than 4 hours per working day

3. Standing

0 normal, can stand for roughly 1 consecutive hour (spectator at sports events)
1 slightly limited, can stand for roughly 15-30 consecutive minutes (waiting in line for theme park attraction)
2 limited, can stand for roughly 5-15 consecutive minutes (washing up)
3 very limited, can stand for less than 5 consecutive minutes (brushing teeth)

4. Standing during work

0 normal, if required, can stand for almost the whole working day (sales jobs, production line jobs)
1 slightly limited, if required can stand for half the working day (roughly 4 hours)
2 limited, if required can stand for a limited part of the working day (roughly 1 hour)
3 very limited, can stand for less than 30 minutes per working day

5. Active kneeling or squatting

0 normal, can perform activities kneeling or squatting for at least 5 minutes (gardening)
1 limited, can perform activities for less than 5 consecutive minutes (cleaning kitchen cupboard door)

6. Active bending and/or twisting

0 normal, can perform activities bending or twisting for at least 5 minutes (sweeping steps)
1 limited, can perform activities bending or twisting for less than 5 consecutive minutes (tying shoelaces)

7. **Active above shoulder level**

0 normal, can perform activities above shoulder level for at least 5 minutes (hanging up curtains)
1 limited, can perform activities bending or twisting for less than 5 consecutive minutes (changing a light bulb)

8. **Keeping head in a certain position during work**

0 normal, if required, can keep head in a certain position for almost the whole working day (screen work, quality control)
1 slightly limited, if required, can keep head in a certain position for half of the working day (roughly 4 hours)
2 limited, if required, can keep head in a certain position for a limited part of the working day (roughly 1 hour)
3 very limited, can keep head in a certain position for less than thirty minutes per working day

9. **Changing position**

0 normal, no specific sequence of different positions required
1 specific requirements of various positions required, i.e. ....

10. **Other limitations to static movement**

0 normal, no other specific limitations in daily life
1 other specific limitations, i.e. ....

11. **Specific conditions for static movement at work**
(are static movements at work dependent on specific conditions because of the said limitations or the client's compensatory behaviour?)

0 no, there are no specific conditions for static movements at work
1 yes, there are specific conditions static movements at work, i.e. ....

Explanation: *see medical insurance physician's report*

HEADING VI: WORKING HOURS

1. Periods in a day (24 hours)

0 normal, if required can work at any hour of the day, night included
1 limited, cannot work nights (00.00 - 06.00)
2 limited, cannot work evenings (18.00 - 24.00)

2. Hours per day

0 normal, can work at least 8 hours per day
1 somewhat limited, cannot work on average more than 8 hours per day
2 slightly limited, cannot work on average more than roughly 6 hours per day
3 limited, cannot work on average more than roughly 4 hours per day
4 extremely limited, cannot on average work more than roughly 2 hours per day

3. Hours per week

0 normal, can work an average of at least 40 hours per week
1 somewhat limited, can work an average of roughly 40 hours per week
2 slightly limited, can work an average of roughly 30 hours per week
3 limited, can work an average of roughly 20 hours per week
4 extremely limited, can work an average of roughly 10 hours per week

4. Other limitations with regard to working hours

0 normal, there are no other specific limitations regarding working hours
1 other specific limitations, i.e. ....

Explanation: *see medical insurance physician's report*
