# Supplementary material for: A Clinically Integrated Post-Graduate Training Programme in Evidence-Based Medicine versus ‘No Intervention’ for Improving Disability Evaluations: A Cluster Randomised Clinical Trial
Source: PLoS One. 2013 Mar 1;8(3):e57256. doi: 10.1371/journal.pone.0057256 (PMC3585805; doi:10.1371/journal.pone.0057256)
Supplement: Appendix S2 — List of quality indicators with criteria for the primary outcome ‘evidence based disability evaluation’. (PDF) [file pone.0057256.s002.pdf]

Appendix S2: List of quality indicators with criteria for the primary outcome 'evidence based disability evaluation'.

Quality Indicator (QI)	Score	Criteria
1. The presence of evidence	Yes (1)	Score Yes if the information reported refers to specific written external information (evidence). *E.g. in the report the following is written (example): "A systematic review on EMDR was found in PubMed" and/or this article was attached to the report*
	No (0)	No evidence reported, or the information in the report reflected the physician's own professional judgment and/or information from a colleague
2. A discernible EBM question	Yes (1)	Score Yes if a medical question was formulated and/or in the report an answer is given to a medical question: Therapeutic, Etiologic, Prognostic, Diagnostic, Background, Incidence, Risk or Other*: ………………………………………………………… **for example: "EMDR has been found to have a favorable effect", which is an answer to a therapeutic question.*
	No (0)	No question and/or answer.
3. The presence of an adequate search strategy	Yes (1)	Score Yes if at least two (specific) search terms were formulated from different PICO domains, or from one PICO domain in combination with the study design, e.g. *"PTSD" is mentioned in the P domain and "EMDR" in the I domain, or "PTSD" in the P domain and RCT design studies.*
	No (0)	Score No if no adequate search strategy is used.
	N/A(-)	N/A: the QI is removed from the denominator (see the last line of this table) if reference is made to the use of a (desktop) guideline, or desktop handbook Work and Workload "Handboek Arbeid en belastbaarheid".
4. A clear EBM source	Yes (1)	Score Yes if the source that was used is verifiable, e.g. (CBO) guideline anxiety disorders, "Handboek Arbeid en Belastbaarheid" (Handbook Work and Workload), title of the article and name of journal, and/or the EBM source is added to the report, e.g. an article on treatment of PTSD with EMDR.
	No (0)	Score No if a clear EBM source is absent or formulated too general, for example when reference is made to "a guideline", " a book", or "an article".
5. An evaluation of the quality of the evidence	Yes (1)	Score Yes if a clear identifiable guideline (e.g. SIP guideline) or Cochrane review was used<br>OR Score Yes if one or more of the following five aspects are mentioned:<br>* the <u>quality</u> of the EBM source (for instance an article), is shown by substantive good use of one of the following concepts: appropriate study design; adequate blinding; randomization; comparability with the control group; follow-up was long enough; the power/sample size was adequate.<br>* the <u>applicability/relevance</u> of the evidence for this patient, which is shown by substantive good use of one of the following concepts: comparability of the specific patient with the research population; feasibility of the intervention; patient or illness oriented outcome; the information is up to date/of a recent date.<br>* the l<u>evel</u> of the evidence that was used, e.g. level 1 evidence exists for the effect of EMDR on PTSD symptoms.<br>* the <u>size</u> of the findings/estimates, which is shown by substantively good use of the concept "clinical relevance" with an example of effect size, such as specificity, sensitivity, LR, NNT, RR, ARR, PPV, NPV.<br>* the <u>(statistical) significance</u> of the findings, using at least two of the following concepts adequately: p-value, confidence interval, power, precision of estimates, type 1 or 2 errors.
	No (0)	Score No if no reference is made to quality of the evidence or the statement is too general, or without explanation, for example "the validity of the research is good".
6. The actual use of evidence in the underpinning of the conclusion	Yes (1)	Score Yes if the evidence that was found is referred to in the underpinnings of the conclusion, explaining the evidence is 'weighted'. This should be in a distinct section of the report, such as below the caption 'medical judgment'. *For example: "as the (CBO) guideline for anxiety disorders shows, EMDR is an important treatment method for PTSD, which is why I recommend this client to follow an EMDR treatment, in consultation with his general physician.*
	No (0)	Score No if no evidence is referred to in the underpinnings of the conclusion.
